# Controlled self-assembly of plant proteins into high-performance multifunctional nanostructured films

**DOI:** 10.1038/s41467-021-23813-6

**Published:** 2021-06-10

**Authors:** Ayaka Kamada, Marc Rodriguez-Garcia, Francesco Simone Ruggeri, Yi Shen, Aviad Levin, Tuomas P. J. Knowles

**Affiliations:** 1 Yusuf Hamied Department of Chemistry, University of Cambridge, Cambridge, UK; 2 Xampla Ltd, Cambridge, UK; 3 Laboratory of Organic Chemistry, Wageningen University, Wageningen, The Netherlands; 4 Laboratory of Physical Chemistry, Wageningen University, Wageningen, The Netherlands; 5 School of Chemical and Biomolecular Engineering, University of Sydney, Sydney, NSW Australia; 6 Cavendish Laboratory, University of Cambridge, Cambridge, UK

**Keywords:** Biomaterials - proteins, Self-assembly, Nanofabrication and nanopatterning, Nanostructures

## Abstract

The abundance of plant-derived proteins, as well as their biodegradability and low environmental impact make them attractive polymeric feedstocks for next-generation functional materials to replace current petroleum-based systems. However, efforts to generate functional materials from plant-based proteins in a scalable manner have been hampered by the lack of efficient methods to induce and control their micro and nanoscale structure, key requirements for achieving advantageous material properties and tailoring their functionality. Here, we demonstrate a scalable approach for generating mechanically robust plant-based films on a metre-scale through controlled nanometre-scale self-assembly of water-insoluble plant proteins. The films produced using this method exhibit high optical transmittance, as well as robust mechanical properties comparable to engineering plastics. Furthermore, we demonstrate the ability to impart nano- and microscale patterning into such films through templating, leading to the formation of hydrophobic surfaces as well as structural colour by controlling the size of the patterned features.

## Abstract (web summary)

Green use of plant derived proteins in functional materials has been limited by inefficient methods to control micro and nanoscale structure. Here, the authors use nanoscale assembly of water-insoluble plant proteins to make meter scale films with comparable properties to conventional plastics.

## Introduction

Naturally derived polymers, or polymers obtained from renewable sources, are actively being explored as alternative building blocks to replace their synthetic counterparts, such as polyethylene terephthalate (PET) and polyvinylchloride (PVC)^1,2^ which continue to be released at vast scales and accumulate in different ecosystems, leading to a global and acute environmental challenge. In this context, polypeptide molecules represent an attractive building block that can be obtained from natural sources, making them suitable for the fabrication of biodegradable materials^3–5^. Proteins possess a propensity toward molecular self-organisation and self-assembly which underpins their roles in a wide range of processes in living systems, including the generation of natural functional materials with remarkable performance^6,7^. To date, the generation of protein-based films through controlled self-assembly has mainly focused on the use of synthetic peptides, natural animal-derived proteins such as silk, β-lactoglobulin, and lysozyme^8–13^ or proteins generated through protein engineering^14,15^. However, the development of protein-based films towards practical applications has remained challenging due to elevated production costs, potential allergenicity, and the environmental impact originating from animal-derived feedstocks^16^.

By contrast, plant-based proteins are promising candidates for generating protein-based films by replacing those derived from animals as they can be sourced in abundance in a sustainable lower environmental impact manner, including as waste products from other industries^17–21^. However, the majority of plant-based proteins are poorly soluble in water, thus generating fundamental challenges for controlling their self-assembly into ordered structures^22–28^. As such, to date, progress has been made by utiliisng complex purification of proteins to selectively extract water-soluble matter, which further limits the process scalability^29–32^.

Here, we describe a processing method to tailor nano and microscale architectures to generate functional plant protein-based flexible films through a multiscale self-assembly approach. This approach exploits the use of acetic acid–water binary mixtures as an environmentally friendly solvent to generate precursor solutions with concentrations as high as 10 w/v% which can then undergo self-assembly as the temperature of the system is lowered. This strategy leads to materials with enhanced intermolecular interactions guided by hydrogen bond formation, allowing their self-assembly into intermolecular β-sheet-rich networks. Crucially, in a second step, solvent removal results in a water-insoluble film with advantageous optical and mechanical properties. This strategy offers new opportunities for plant-based protein processing by overcoming limitations associated with poor aqueous solubility and offers an enhanced level of control of the nanoscale properties of the material. We further extended this approach by combining the protein self-assembly with microlithographic techniques to pattern nano- and microstructures, which present further opportunities for the generation of nano-scaled ordered materials with a wide range of future applications in the fields of materials science and photonics.

## Results

Suitable solvent systems capable of dissolving plant proteins and direct their self-assembly into aggregated structures are required to improve their processability into structured materials. Previous approaches have exploited the use of nonvolatile chaotropic agents to extensively denature proteins, but these often remain in the final product and can greatly affect the final material properties^33–35^. In this work, we exploit the use of binary mixtures of water and acetic acid, alongside exposure to ultrasonication and elevated temperatures, to improve the solubility of a model plant protein feedstock. Acetic acid was chosen due to its ability to enhance the solvation of hydrophobic amino acid residues under aqueous-compatible conditions^36,37^, and ultrasonication treatment was applied in order to disrupt large protein aggregates into smaller particles, as well as destabilizing intermolecular interactions, ultimately promoting the formation of small soluble protein aggregates^28,38–40^. Together with exposure to elevated temperatures, the protein can be fully dissolved and denatured, enabling the formation of new intermolecular interactions in a controlled manner.

**Fig. 1:**
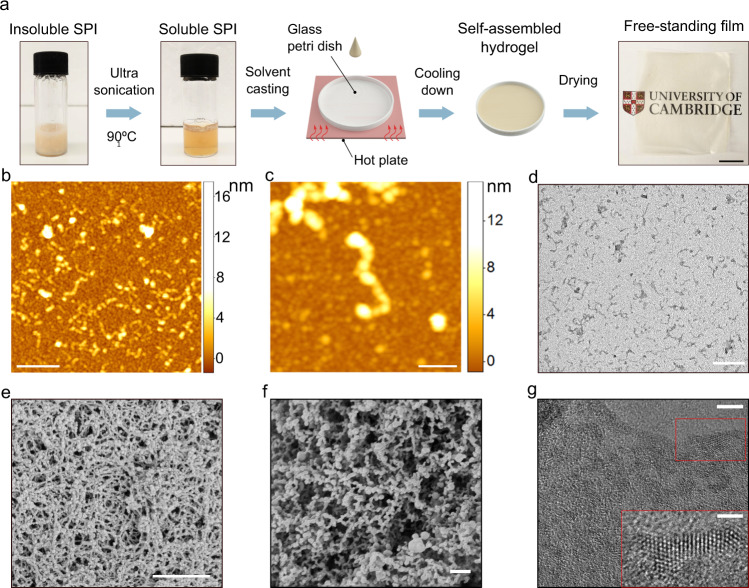
Solvation of plant proteins and generation of films through molecular self-assembly. **a** A translucent aqueous soyprotein isolate (SPI) solution (10 w/v% SPI, 30 v/v% acetic acid) was obtained via ultrasonication treatment at elevated temperature (90 °C) for 30 min. The resultant SPI solution was cast on a pre-heated glass Petri dish. Upon cooling, a translucent hydrogel was formed. Following evaporation of the solvent, a free-standing film was obtained (scale bar 1 cm). **b**–**d** AFM images (**b**, **c**), and TEM image (**d**) of SPI fibrillar aggregates formed through the self-assembly process. Scale bars are 500 nm for **b** and **d**, and 100 nm for **c**. **e** SEM image of SPI hydrogel prepared through supercritical CO_2_ drying. Scale bar is 500 nm. **f** cryo-SEM image of SPI hydrogel. Scale bar is 500 nm. **g** TEM image of β-sheet nanocrystals in dried SPI film. Scale bars are 5 nm for the main image and 2 nm for the inset.

We first utilised this approach to prepare a soy protein isolate (SPI) solution, which was then processed into a film by solvent-casting (Fig. 1a). SPI was chosen as a model plant-based protein since it is one of the most available sources of plant proteins and is available as a by-product of soybean oil production^26^. A colloidal protein slurry was first prepared by dispersing SPI at 10 w/v% protein concentration in a 30 v/v% acetic acid aqueous solution. The slurry was then exposed to ultrasonication treatment and elevated temperature (90 °C) for 30 min, which resulted in the formation of a translucent aqueous solution (Fig. 1a). Turbidity measurements, solubility measurements, dynamic light scattering (DLS), optical microscopy, and transmission electron microscopy (TEM) characterisation revealed that proteins processed in aqueous acetic acid solution have a significantly smaller particle size (29 ± 9.1 nm) compared to a control protein sample dispersed in deionised water (148 ± 68 nm, Supplementary Figs. 2 and 3) as well as higher transparency and solubility (Supplementary Fig. 1).

Through controlled lowering of the temperature of a treated SPI solution, the formation of a self-standing hydrogel was observed. The obtained hydrogel was analyzed using scanning electron microscopy (SEM), where the sample was prepared either under cryogenic (cryo-SEM) or through supercritical CO_2_ drying (see “Methods”). SEM imaging revealed that the proteins self-assembled into a densely packed hydrogel network comprised of fine-stranded aggregates (Fig. 1e, f). By contrast, SPIs prepared without the complete solvation, such as in deionised water at various pH, were not able to form a self-standing hydrogel after the ultrasonication treatment (Supplementary Fig. 4), highlighting that the dissolution of SPI in a binary mixture of water and acetic acid is key for directing the proteins to self-assemble into a hydrogel network.

In order to further observe the micromorphology of the aggregated protein structures formed upon lowering of the temperature, a diluted protein solution (2 w/v% in 30 v/v% acetic acid) was prepared and imaged through TEM and atomic force microscopy (AFM). Microstructure analysis revealed the presence of fibrillar structures with lengths of 100–200 nm and a diameter of 5–10 nm (Fig. 1b, d). By contrast, when the SPI solution was diluted prior to lowering the temperature, fibrillar structures were not observed (Supplementary Fig. 3), a finding which confirms that the self-assembly of protein fibrillar structures takes place through lowering of the temperature. It was further confirmed through TEM and AFM imaging that similar fibrillar aggregates are present in the hydrogel, where the hydrogel was heat-melted at 95 °C and subsequently diluted in 30 v/v% acetic acid for imaging (Fig. 1c and Supplementary Fig. 5).

To obtain spatially uniform films, glycerol was added as a plasticiser (30 w/w% to the total dry mass) after the sonication treatment and the solution was drop-cast onto a glass Petri dish. The petri dish was heated to 90 °C during the casting step, which allowed the uniform distribution of the protein solution on the substrate, and the subsequent formation of a thin hydrogel upon cooling down to room temperature. Finally, the cast hydrogel was allowed to dry at room temperature for 3 days, solidifying into a free-standing film. TEM observation of the dried protein solution revealed that significant quantities of nanocrystalline structures were formed, with overall dimensions of the order of ca. 5–10 nm, a value comparable to analogous structures found in silk. (Fig. 1g)^41–44^. Moreover, the constant spacing of ca. 3.5 Å found is consistent with β-sheet nanocrystals observed in silk structures and other protein-based films^45–48^.

**Fig. 2:**
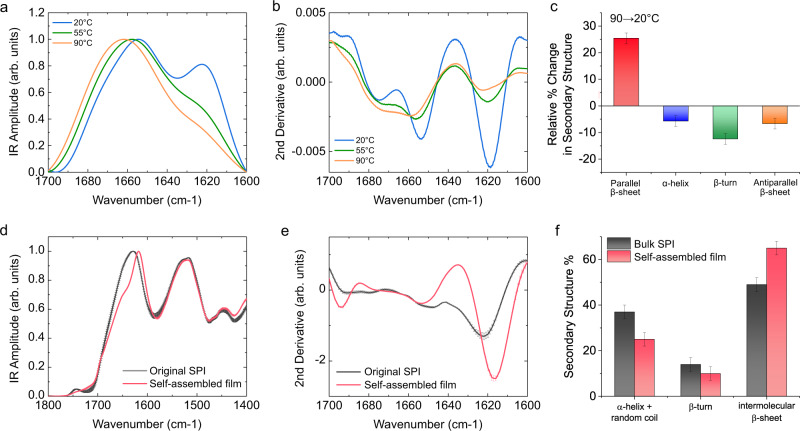
Secondary structure analysis of SPI self-assembly. **a**, **b** ATR-FTIR spectra of SPI solution during cooling down from 90 to 20 °C (**a**) and their second derivatives (**b**). **c** Relative change in the secondary structure during cooling down from 90 to 20 °C. **d**, **e** ATR-FTIR spectra of original SPI powder and the dried self-assembled film (**d**) and their second derivatives (**e**). **f** Quantification of secondary structure content calculated from Amide I band of IR spectra for original SPI powder and the dried self-assembled film. The indicated error bars are the s.d. of the average of three different spectra, each one is co-average of 256 scans.

The self-assembly process during the gelation of a SPI solution upon lowering the temperature from 90 to 20 °C was investigated through attenuated total reflectance Fourier-transform infrared spectroscopy (ATR-FTIR, Fig. 2a). The IR spectrum is characterized by the presence of the Amide I band of protein (1700–1600 cm^−1^), which arises from the vibration of the C=O stretching of the protein backbone, and it is strictly related to the protein secondary and quaternary structural contributions in our sample. The deconvolution and the quantification of the abundance of these structural contributions can be evaluated by calculating the second derivative of the Amide I band, and then integrating the area of the peaks related to the different secondary structural contributions in the spectrum, as previously shown in literature^49–51^. Just after the sonication treatment at 90 °C, the analysis of the IR spectra and their second derivatives showed that proteins possess a low content of intermolecular β-sheet structure and high content of random coil and α-helical secondary structure. Upon cooling down the sample to 20 °C, the content of intermolecular β-sheets gradually increased by 25% (Fig. 2a–c), as well as a significant shift of the Amide I band to lower wavenumbers, indicating the formation and increase of intermolecular hydrogen bonding.

In order to investigate the protein secondary structure and the degree of intermolecular interactions in the SPI film, FTIR and XRD analysis were performed. FTIR analysis of the generated film showed a higher amount of intermolecular β-sheet structure (65%) compared to the original SPI powder (49%) or films prepared without complete solvation of the protein (46%), as an example of a protocol extensively reported in the literature (Fig. 2d–f and Supplementary Fig. 6)^52–55^. X-ray diffraction (XRD) characterization also revealed higher intensity for intermolecular β-sheet (d-spacing value of ~10 Å) in our film compared to the original SPI powder (Supplementary Fig. 7)^56^.

**Fig. 3:**
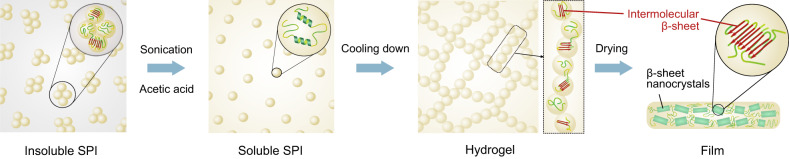
Schematic representation of a proposed mechanism for the self-assembly of SPI. Through the ultrasonication treatment in acetic acid solution, the initially insoluble aggregates are solubilised and unfolded, making them avaialble to form new intermolecular interactions. Upon cooling down, the new intermolecular β-sheets structures are formed. The removal of solvent results in the formation of β-sheet nanocrystals within the film.

Together with FTIR characterization performed during the gelation, the mechanism of self-assembly is proposed as follows (Fig. 3). Originally, SPI has a high content of intermolecular β-sheet (49%) and is poorly soluble in water. Upon exposure to elevated temperature and sonication treatment in aqueous acetic acid solution, the proteins unfold and partially hydrolyse, making them more available to form new intermolecular interactions. Controlled lowering of the solution temperature after the sonication treatment facilitates the protein self-assembly into intermolecular β-sheet-rich fibrillar aggregates, stabilised by stronger hydrogen bonding, leading to the formation of a hydrogel. Further removal of the solvent allows to retain the high content of intermolecular β-sheet structures (65%) and less amount of random or α-helix structure (25%) in the dried film, exhibiting a high degree of crystallinity compared to the initial SPI powder. Interestingly, the dissolution of proteins in acetic acid solution and subsequent formation of hydrogel was found to produce similar results with other plant protein feedstocks, such as pea protein isolate (Supplementary Fig. 8).

**Fig. 4:**
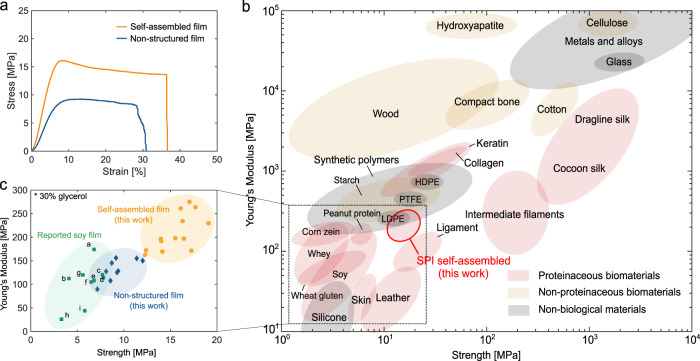
Mechanical properties of the self-assembled SPI film. **a** Representative stress–strain curves for the dried nonstructured and the dried self-assembled films. **b** Mechanical properties of self-assembled SPI film in comparison to previously reported biomaterials, engineered materials^6,71,72^, and plant-based materials (see Supplementary Table 2 for references). **c** Zoomed-in graph for self-assembled (orange), nonstructured (blue), and previously reported SPI films (green, the details of references are in Supplementary Table 3).

Moreover, tensile testing of the dried SPI films revealed that the mechanical behaviour is significantly improved through the self-assembly process. The self-assembled film containing 30 w/w% glycerol (relative to the total dry mass) achieves high tensile strength (15.6 ± 2.07 MPa) and Young’s modulus (209 ± 39.1 MPa, Fig. 4a and Supplementary Fig. 9). The addition of glycerol provides options to tailor the mechanical performance from 483 ± 58.4 MPa to 92.7 ± 25.3 MPa for Young’s Modulus and 25.0 ± 3.49 MPa to 6.18 ± 0.98 MPa for tensile strength when the concentration of glycerol was varied from 20 to 40 w/w% (Supplementary Figs. 10 and  11). The tensile strength and Young’s modulus of the self-assembled film with 30% glycerol were moreover found to be higher than values for the nonstructured film with 30% glycerol (9.30 ± 1.53 MPa and 131 ± 22.6 MPa for tensile strength and Young’s Modulus, respectively, Fig. 4a and Supplementary Fig. 9) or values for reported SPI films (Fig. 4c, with 30% glycerol) and are comparable to engineered plastic materials, such as low-density polyethylene (LDPE) and polytetrafluoroethylene (PTFE) (Fig. 3b). Importantly, in previously reported SPI films, mechanical reinforcers such as cellulose or chitin have been extensively used to improve the mechanical properties^57,58^. By contrast, the self-assembled SPI film exhibits similar mechanical performance without using any reinforcers (Supplementary Fig. 12 and Supplementary Table 4) owing to a high degree of intermolecular hydrogen bonding interactions obtained through self-assembly.

**Fig. 5:**
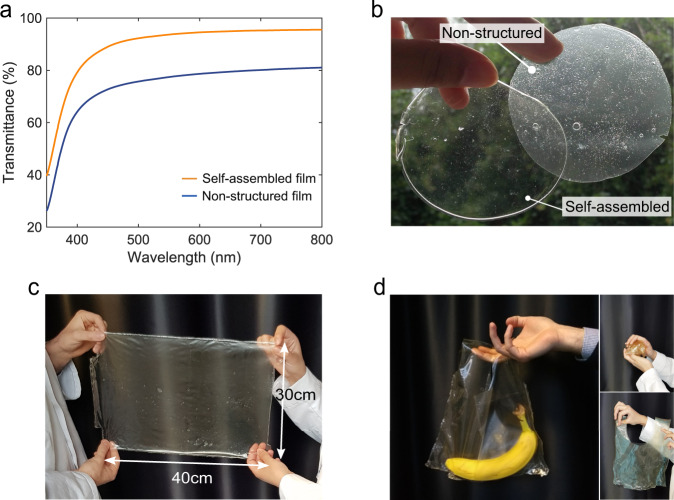
Optical appearance of self-assembled films. **a** UV–vis spectra of nonstructured (blue) and self-assembled films (orange). **b** Optical image of nonstructured (right) and self-assembled (left) SPI films. **c** Photograph of a 30 × 40 cm film fabricated through large-scale processing. **d** Carrying bag generated by thermal welding.

The dissolution of SPI further facilitated the formation of a highly transparent film (Fig. 5a, b), compared to conventional SPI films generated from insoluble SPI (referred as nonstructured film). UV–vis spectroscopy analysis showed that the transmittance at 550 nm wavelength is as high as 93.7% for the self-assembled film, which is significantly superior to a conventional nonstructured SPI film prepared under alkaline conditions, found to be 77.4%. The transparency of the self-assembled film was found to be higher than the ones reported in the literature for SPI films (Supplementary Table 1).

The formation of thin films from self-assembled protein structures has previously been reported for a variety of proteins, such as lysozyme, β-lactoglobulin, whey protein, silk fibroin, and recombinant proteins^8–15^. However, low production yields and extensive purification steps challenge the scalability for the generation of self-assembled materials from such feedstocks, with production costs exceeding $100/kg^59,60^. On the other hand, refined plant-based protein feedstocks, such as soy or pea protein isolates (80–90% w/w protein content) are globally produced at a large scale and can be obtained at a much lower cost ($3.5–4.6/kg). The method described in this work does not require the use of high concentrations of chaotropic agents or organic solvents and exploits the use of water and acetic acid as a cost-efficient and environmentally friendly alternative, which enables the production of functional protein thin films at competitive production costs. Also, ultrasonication is widely used in food processing and thus practically available at industrial scale^61,62^. As a demonstration of the scalability of the process, we fabricated a 30 × 40 cm SPI film (Fig. 5c). Interestingly, we found that these films can be structured into three-dimensional shapes through thermal welding, where the edges of films are melted when pressed together at high temperatures. As an example, two SPI films were sealed by thermal welding to form a carrying bag (Fig. 5d). In addition, we tested another commercial SPI feedstock obtained from different manufacturers (characterizations of these SPIs are included in Supplementary Fig. 13) and confirmed the dissolution of SPI and subsequent self-assembly, leading to a film with similar mechanical properties.

**Fig. 6:**
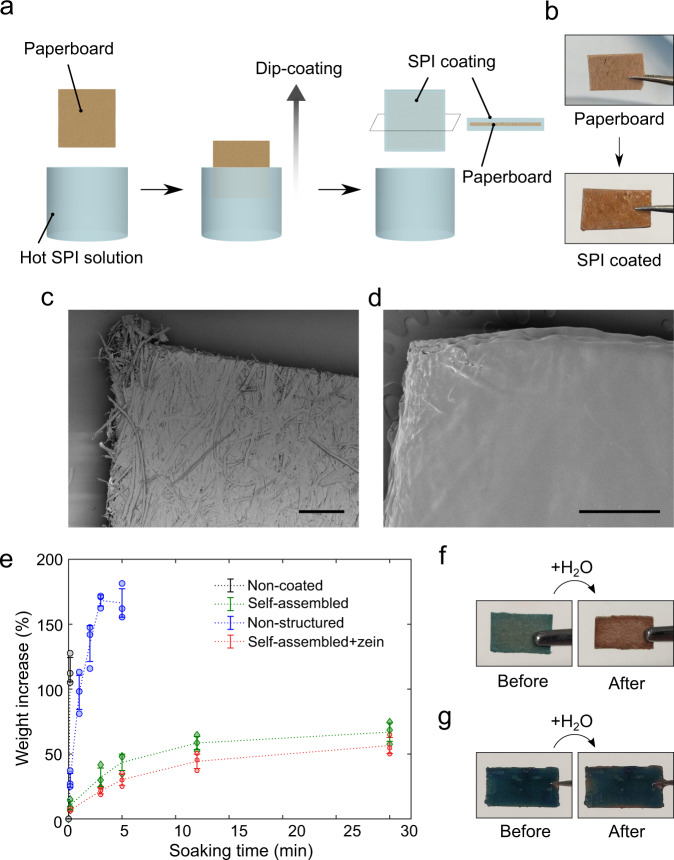
Plant protein film for coating with barrier function. **a** Schematic illustration of the coating process. A piece of paperboard was dipped in the SPI solution and pulled out slowly, leading to the formation of a gel coating its surface. The paperboard was allowed to dry at room temperature to achieve an anhydrous thin layer of coating. **b** Optical images of paperboards before and after SPI coating. **c**, **d** SEM images of paperboard without (**c**) and with (**d**) SPI coating. Scale bars represent 500 μm. **e** Water uptake of the treated and untreated paperboards measured over 30 min studied through gravimetry. The indicated error bars represent the s.d. of the average of three independent measurements. **f**, **g** Colour changes of CoCl_2_-stained paperboards before and after immersing in water for 5 s (Supplementary Movie 1). The paperboard was prepared without (**f**) and with (**g**) SPI coating, respectively.

We further investigated the potential of self-assembled SPI films for coating applications. Due to the sol–gel transition that a concentrated SPI solution undergoes upon cooling down, a thin film can be formed on a substrate via a one-step dip-coating process. By gradually withdrawing the substrate from the heated SPI solution and subsequent air-drying at room temperature, the deposition of a SPI hydrogel is achieved, which can be further solidified to form a coating on the substrate surface (Fig. 6a). We explored the barrier properties imparted by this coating on a composite material consisting of paperboard, which was used as a substrate and coated by an aqueous SPI solution (Fig. 6b–d). Water absorption measurements revealed that the SPI coating reduces the water uptake of paperboard significantly (Fig. 6e). The slower diffusion of water through the SPI coating was also confirmed by the colorimetric test where cobalt chloride was used as humidity sensor (Fig. 6f, g and Supplementary Movie 1)^63^. The water barrier properties of the coating can be further improved by the addition of further hydrophobic proteins. To demonstrate this property, the dip-coating process can be further integrated with another hydrophobic plant protein, corn zein. Only a small amount of zein (0.5 w/v%) significantly further reduced the substrate water uptake (Fig. 6e), showing great potential to tailor the coating functionality. In addition, characterization of the film’s oxygen permeability revealed advantageous gas permeability modulating properties comparable to that of PVC and oriented polyethylene terephthalate (OPET)^64^. These results demonstrate that the sol–gel transition of self-assembled SPI together with its mechanical properties enable a simple and effective dip-coating process, which could be generally applied for coating applications.

**Fig. 7:**
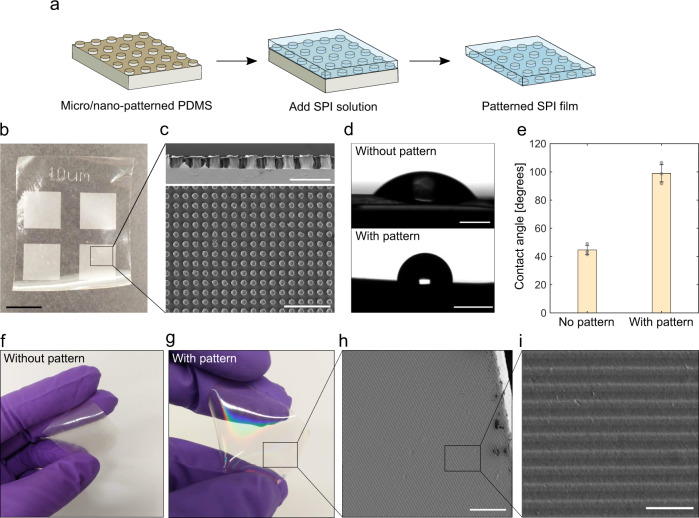
Micro- and nanopatterning of plant protein film. **a** Schematic illustration of the soft lithography process to pattern SPI film. **b** Optical image of the micro-patterned film. Scale bar represents 5 mm. **c** SEM image of the side view (top) and top view (bottom) of patterned micropillars. Scale bars represent 25 μm (top) and 100 μm (bottom). **d** Optical images of a water droplet on the film without (top) and with (bottom) micropillars. Scale bars represent 1 mm. **e** Water contact angles of the films, showing an increase of hydrophobicity in the patterned film. The indicated error bars are the s.d. of the average of three independent measurements. **f**, **g** Optical image of non-patterned (**f**) and nano-patterned photonic film (**g**). **h**, **i** SEM image of the nano-patterned film. Scale bars represent 20 μm (**h**) and 2 μm (**i**).

Furthermore, we combined the fabrication of SPI film with lithographic techniques to expand the functionalities derived by the protein self-assembly. Nano- and micro-patterning through soft lithography techniques have been used to engineer small-scale components useful for a wide range of applications, such as waveguides and diffraction gratings^65–67^. To explore the possibility of various features to be patterned on SPI films, two sets of lithographic substrates (masters) were fabricated to allow microscale features to be stamped on the surface of the film. The first master was fabricated on polydimethylsiloxane (PDMS) through standard UV–soft-lithographic techniques^68^. The photomask was generated such that the negative pattern of micropillars were arranged periodically. The film-forming SPI solution was poured into the PDMS template and allowed to self-assemble and solidify as previously described (Fig. 7a). This process led to the formation of a film with an 5 × 5 mm patterned area (Fig. 7b). SEM images of the patterned area show micropillars of ca 10 μm in diameter and ca 15 μm in height (Fig. 7c). One of the most appealing features of such microstructure is an increase of hydrophobicity in a similar format to the hydrophobic surfaces evolved in nature^69^. Indeed, the micropillars printed on SPI film led to an increase of water contact angle from 45 to 99° (Fig. 7d, e). Such microstructures would give a further range of utility such as immobilization of particles, which could be used as drug patches^70^.

For the second master, nanometre-scale periodic grooves were patterned using an optical disc (digital versatile disc, DVD) as a template. In contrast to non-patterned film, the patterned film shows iridescence due to the diffraction of incident light induced by the patterned nanostructure (Fig. 7f, g). Upon closer inspection using SEM imaging, the patterned surface exhibits a periodic grafting with the measured centre-to-centre distance of ca 750 nm (Fig. 7h, i), consistent with the nanostructure in the optical disc used as a template (740 nm).

## Discussion

The wide range of natural high-performance materials that are generated through multi-scale self-assembly of protein molecules have inspired us to devise a strategy for the scalable generation of protein-based materials. Plant-based proteins are attractive sources for protein-based materials due to the possibility to obtain such feedstocks from sustainable sources at comparatively low cost. However, the low water-solubility of these proteins challenges their direct assembly without the need for modifications and complex purification steps. In this work, we present an approach for overcoming this challenge in generating scalable plant-based protein materials by devising a strategy to control their molecular assembly in aqueous-based solutions. We demonstrate the generation of meter- -scale robust films with plastic-like properties without the requirement for any covalent cross-linkers, toxic solvents, or non-biodegradable materials or products. This approach not only provides a facile route toward multiscale self-assembled structures with high transparency and favourable mechanical properties, but further extends their functionalities by tuning the microscale structures. These results open a route toward scalable self-assembly of plant-based proteins, and offer opportunities for nanostructured protein-based films to be used in a range of applications that currently rely on synthetic materials systems.

## Methods

### Fabrication of plant protein film

Soy protein isolate (SPI) was purchased from MP Biomedicals (purity 92%) and used without further purification. In total, 500 mg of SPI was dispersed in 5 mL of 30% v/v acetic acid aqueous solution and shaken well until a turbid and highly viscous dispersion was obtained. The dispersion was then sonicated using an ultrasonic homogenizer (Bandelin, HD2070, 70 W) for 30 min (40% amplitude, pulse durations of on-time 0.7 s and off-time 0.3 s). Following sonication, glycerol (≥99.5%, Sigma-Aldrich) was added as a plasticizer at various concentrations (125 mg, 214 mg, and 333 mg for 20, 30, and 40 w/w% glycerol, respectively), and the solution was sonicated for an additional minute. The concentration of glycerol was determined by mass with respect to the total mass of SPI and glycerol. The hot liquid solution was immediately cast on a 7-cm glass Petri dish pre-heated at 90 °C. The cast solution was then dried for 3 days at room temperature (19–22 °C) and ambient humidity (typically 30–50% RH). The dried film was then peeled off from the petri dish and stored at room temperature until further use.

As a control experiment, films have were prepared in alkaline conditions:^52–55^ 10 w/v% SPI was dispersed in an alkaline aqueous solution adjusted to pH = 10 with NaOH and heated for 30 min at 95 °C in a water bath (referred as nonstructured film). 30 w/w% glycerol was then added, and the hot liquid was cast on a glass Petri dish and dried for 3 days.

To produce a large film, 60 mL of 10 w/v% protein dispersion was prepared in 30 v/v% acetic acid and sonicated for 30 min (Bandelin HD4200). The obtained transparent solution was cast on a 30 × 40 cm oven tray pre-heated at 110 °C and dried for 3 days at room temperature in a fume hood.

### Characterization of SPI supramolecular aggregates

AFM 3-D maps and TEM micrographs were obtained using Park Systems NX10 and Thermo Scientific FEI Talos F200X G2 TEM, respectively. AFM was performed on mica substrates in non-contact mode and equipped with a silicon tip (PPP-NCHR, 5 Nm^−1^) with a nominal radius <10 nm. The protein solution was deposited on a fresh mica surface and washed with Milli-Q water after 15 min of incubation at room temperature. The raw images were flattened using the built-in software (XEI, Park System, South Korea). For TEM, the solution was deposited on TEM grids (C400Cu, EM resolutions), stained with 2% uranyl acetate. For the imaging of supramolecular aggregates, the sonicated protein solution after cooled down was diluted in 30 v/v% acetic acid to achieve protein concentration of 0.1 w/v%. For imaging of proteins before self-assembly, a dilution step was performed immediately after the sonication treatment using a pre-heated 30 v/v% acetic acid solution and quickly deposited on the TEM grids to avoid the protein self-assembly.

In order to characterize the solubility of SPI, SPI dispersions were prepared in four different solvents (ultrapure water, pH = 10 NaOH adjusted solution, pH = 2 HCl adjusted solution, and 30 v/v% acetic acid solution) and treated either by heating for 30 min at 95 °C in a water bath or by ultrasonication for 30 min. The turbidity measurements were performed using a microplate reader (FLUOstar-BMG labtech), and absorption at 600 nm was recorded. In order to determine the protein solubility, 2 w/v% SPI dispersions were centrifuged at 12,000 × *g* for 15 min at 20 °C, and the protein concentration of the supernatant was measured by weighing the mass after evaporation of the solvent.

Particle size and zeta potential were measured using Zetasizer (Malvern Panalytical). Protein dispersions were quickly diluted to 0.1 w/v% after the heating or sonication treatments to avoid protein self-assembly, and the measurements were performed immediately after. For zeta potential measurements, the pH was adjusted using either 1 M HCl or 1 M NaOH.

The protein molecular weight distribution was determined using SDS polyacrylamide gel electrophoresis (NuPAGE Novex 4–12% Bis–Tris gels and NuPAGE MES SDS running buffer). SPI samples were diluted to 1 mg/mL in Milli-Q water. Zein samples were diluted to 1 mg/mL in 70% ethanol. All protein samples were reduced by β-mercaptoethanol. Gels were stained using Instant Blue stain (Sigma-Aldrich).

### Characterization of hydrogel and film

SEM images were obtained using a FEI Verios 460 scanning electron microscope. For critical point drying, SPI hydrogels were gradually dehydrated in 50%, 75%, and 100% ethanol for 24 h. Samples were transferred to microporous specimen capsules (78-µm pore size, Agar Scientific) soaked and part-filled with absolute ethanol to prevent the sample from drying during transfer. Then, samples were dried using a Quorum E3100 critical point dryer using four to five flushes with liquid CO_2_ and at least 15 min of incubation between each flush. Samples were mounted on aluminium SEM stubs using conductive carbon sticky pads (Agar Scientific) and coated with 15-nm iridium using a Quorum K575X sputter coater. Cryo-SEM was performed with a Quorum PP3010T cryo-transfer system. Secondary electron images were acquired at 1–2 keV accelerating voltage and 25 pA probe current using a Through-Lens detector in full immersion mode.

In order to observe the β-sheet nanocrystals through TEM, 2 w/v% SPI solution in 30 v/v% acetic acid was prepared through the sonication treatment and deposited on TEM grids. The grid was allowed to dry in order to form a thin film and stained with 2% uranyl acetate. TEM micrographs were obtained using Thermo Scientific FEI Talos F200X G2 TEM.

Fourier-transform infrared (FTIR) spectroscopy data were collected using FTIR VERTEX 70 spectrometer (Bruker) with a diamond (air measurements) and ZnSe (hydrogel measurements) attenuated total reflection (ATR) element. The hydrogel samples were used without further pre-treatment and were loaded into the FTIR holder and analysed by subtracting the same concentration of acetic acid as a reference. The atmospheric compensation spectrum was subtracted from the original FTIR spectra and a secondary derivative was applied for further analysis. The resolution was 4 cm^−1^ and all spectra were processed using Origin Pro software. The spectra were averaged (3 spectra with 256 co-averages), smoothed applying a Savitzky-Golay filter (2nd order, 9 points) and then the second derivative was calculated applying a Savitzky-Golay filter (2nd order, 11 points). The relative secondary and quaternary organization was evaluated by integrating the area of the different secondary structural contributions in the Amide band I, as previously shown in literature^49–51^. The error in the determination of the relative secondary structure content is calculated over the average of at least three independent spectra and it is <5%.

XRD data were collected on a Panalytical X’Pert Pro diffractometer using non-monochromated CuKα radiation with a step size of 0.0167° and a total collection time of 40 min. The samples were gently ground where possible and distributed on a glass sample holder. A background measurement was taken under the same conditions using the glass sample holder only.

The tensile test was performed with Tinius Olsen H25KS using 250 N load cell. The film was cut into 5-mm-width slips and the thickness of the film was measured for each sample using a digital calliper. The measurements were performed at a loading speed of 2 mm/min. All tests were repeated at least nine times. UV–vis optical transmittance was measured using Cary 500 UV–vis spectrometer.

The oxygen permeability measurement was carried out by Smithers Rapra and Smithers Pira Ltd (UK). The oxygen gas transmission rate through the film was determined at 23 ± 2 °C, 50 ± 5% relative humidity using Oxtran 2/21 apparatus. The sample was mounted to create a membrane between two chambers. Both chambers were initially flushed with a carrier gas (2% hydrogen in nitrogen), and then oxygen was flushed through the outer chamber. The sensor was then activated to detect the amount of oxygen that had permeated through the sample, and measurements were taken over the course of several hours until the system had reached equilibrium. The sample was subjected to 12- h conditioning period, then tested using converge mode whereby equilibrium is achieved when the difference between the current transmission rate and the transmission rate obtained 5 h previously is <1%. Four replicates were tested to 100% oxygen with the outer surface of the film facing the oxygen. The oxygen permeability was determined to be 2012 ± 820 cc‧μm/m^2^‧day‧atm.

### SPI coating of paperboard

A paperboard (thickness ca 0.75 mm) was cut into ca 7 × 13 mm pieces. 10 w/v% soy protein solution was prepared in 30 v/v% acetic acid using ultrasonication in the absence of a plasticizer. A piece of paperboard was immersed into the protein solution immediately after the sonication treatment. After dipping the paperboard in the solution for 10 s, the paperboard was taken out from the solution slowly and dried at room temperature for 3 days.

In order to measure the water uptake, the paperboard was immersed in 10 mL of pure water and the weight increase was measured periodically at different time intervals from 10 s to 30 min. The initial weight of the paperboard was measured before the immersion and used for normalization of the weight increase. Nonstructured SPI was coated in the same manner in the absence of a plasticizer. To prepare a zein-containing soy protein solution, 0.5 w/v% zein (Acros Organics) and 10% w/v soy protein were mixed with 60 v/v% acetic acid and sonicated for 30 min.

### Surface patterning of the film

A soft-lithographic replica moulding approach was developed to pattern micro- and nanostructures on the SPI film surface, a. An array of square microstructures was first patterned on a 3-inch silicon wafer via a photolithographic technique. In all, 1 ml of SU-8 3025 photoresist (MicroChem) was spin-coated on a 3-inch silicon wafer. The coated wafer was then soft-baked at 95 °C for 15 min. A photolithographic mask comprised of an array of micropillars was placed on the coated wafer and exposed to UV light for 25 s. After 5 min of postexposure bake at 95 °C, the photoresist was developed using propylene glycol methyl ether acetate. After cleaning the surface of the wafer with isopropanol, a mixture of polydimethylsiloxane (PDMS) and curing agent (Sylgard 174, Dow Corning) in a 10:1 ratio was poured on the wafer and cured at 65 °C for 1 h. The cured PDMS was peeled off from the wafer and used as a negative pattern for the soy film.

To make photonic films from the nano-pattern of a DVD, the outer plastic layer was carefully removed from the disc and the remaining middle layer was collected. PDMS was poured onto the collected part of DVD and cured for 1 h at 65 °C. The patterned PDMS was peeled from the layer of DVD, and the sonicated soy protein solution was cast on the PDMS.

Before casting protein solution on the PDMS template, the PDMS slab was degassed in pure water. The sonicated soy protein solution containing 30 w/w% glycerol was cast onto the PDMS substrate and allowed to dry to form the patterned film.

The contact angle of the micro-patterned film was measured using FTA1000B (First Ten Angstroms). A small water droplet was placed on the protein film fixed at the horizontal plate and the optical image of the droplet was taken immediately. The contact angle was calculated using FTA20 (First Ten Angstroms). The measurement was repeated three times using different locations on the films. The surface of the micro-patterned film was observed using TESCAN MIRA 3 FEG-SEM sputter-coated with 10-nm platinum.

## Supplementary information

Supplementary Information

Description of Additional Supplementary Files

Supplementary Movie 1

## Data Availability

All relevant data are included within this article and its Supplementary Information files. Any additional information is available from the corresponding author on reasonable request.
