# Supplementary material for: Controlled self-assembly of plant proteins into high-performance multifunctional nanostructured films
Source: Nat Commun. 2021 Jun 10;12:3529. doi: 10.1038/s41467-021-23813-6 (PMC8192951; doi:10.1038/s41467-021-23813-6)
Supplement: Supplementary file 1 — Supplementary Information [file 41467_2021_23813_MOESM1_ESM.pdf]

Supplementary information for

Controlled self-assembly of plant proteins into high-performance multifunctional nanostructured films

Ayaka Kamada[1†], Marc Rodriguez-Garcia[1,2†], Francesco Simone Ruggeri[1,3-4], Yi Shen[1,5], Aviad Levin[1] and Tuomas P. J. Knowles[1,6*]

Affiliations:

[1]Yusuf Hamied Department of Chemistry, University of Cambridge, Lensfield Road, Cambridge CB2 1EW, UK

[2]Xampla Ltd, Cambridge Science Park Road, CB4 0FW, Cambridge UK

[3]Laboratory of Organic Chemistry, 6703 WE, Wageningen University, the Netherlands

[4]Laboratory of Physical Chemistry, 6703 WE, Wageningen University, the Netherlands

[5]School of Chemical and Biomolecular Engineering, University of Sydney, NSW 2006, Australia

[6]Cavendish Laboratory, University of Cambridge, CB3 0FE, Cambridge UK

*Correspondence to: tpjk2@cam.ac.uk

†These authors contributed equally to this work.

Supplementary Figures

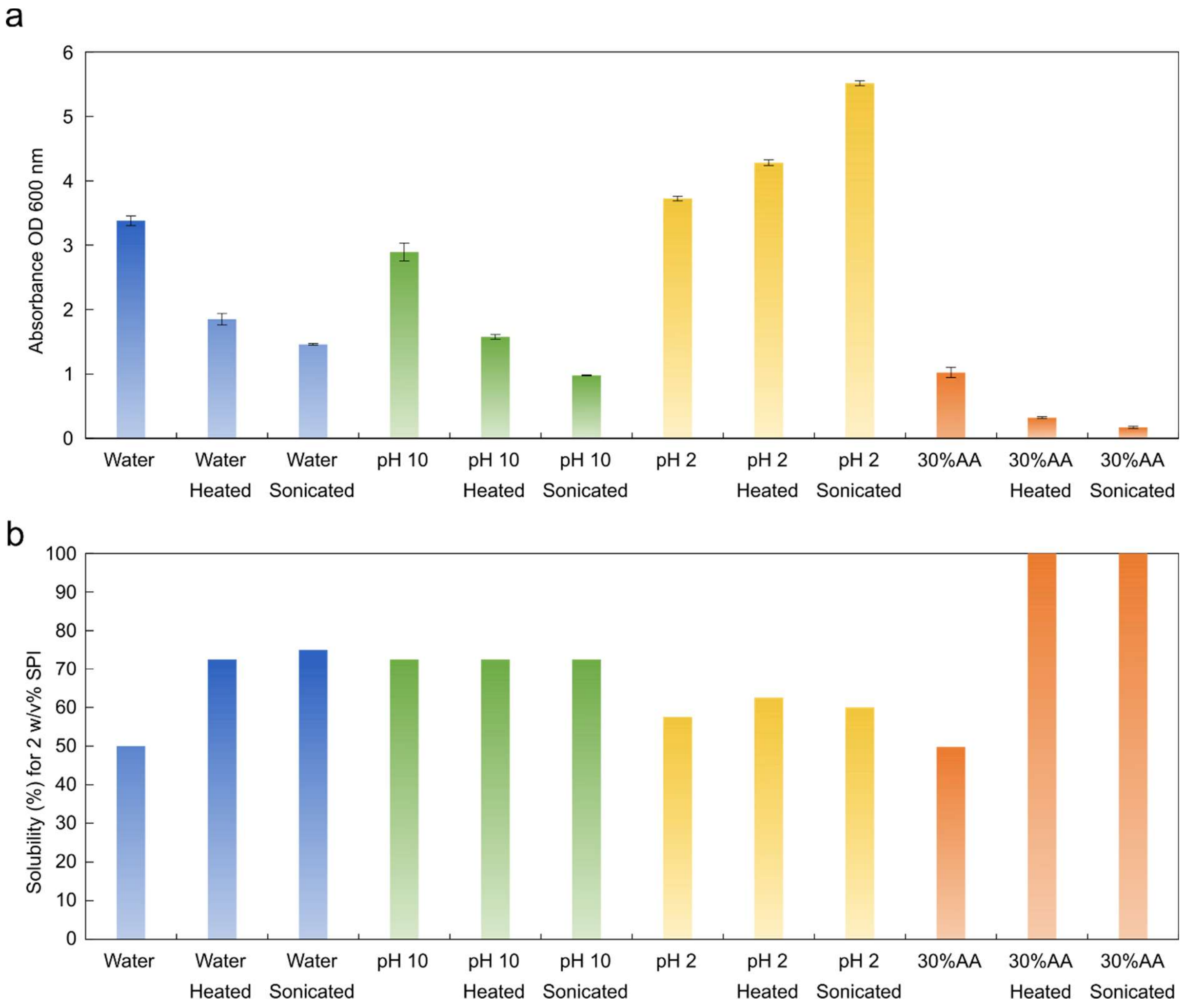

Supplementary Figure 1 Turbidity and solubility measurements for SPI solutions prepared in water, pH 10 NaOH, pH 2 HCl or 30 v/v% acetic acid and treated with three different methods, (i) non-treated, (ii) heated for 30 min at 95 °C (noted as heated), and (iii) sonicated for 30 min (noted as sonicated). **a,** Turbidity measurement for 2 w/v% SPI solutions performed at 600 nm wavelength. Data are shown as mean ± s.d.; n = 3 independent experiments. **b,** Solubility measurement for 2 w/v% SPI determined through centrifugal separation of non-solubilized proteins.

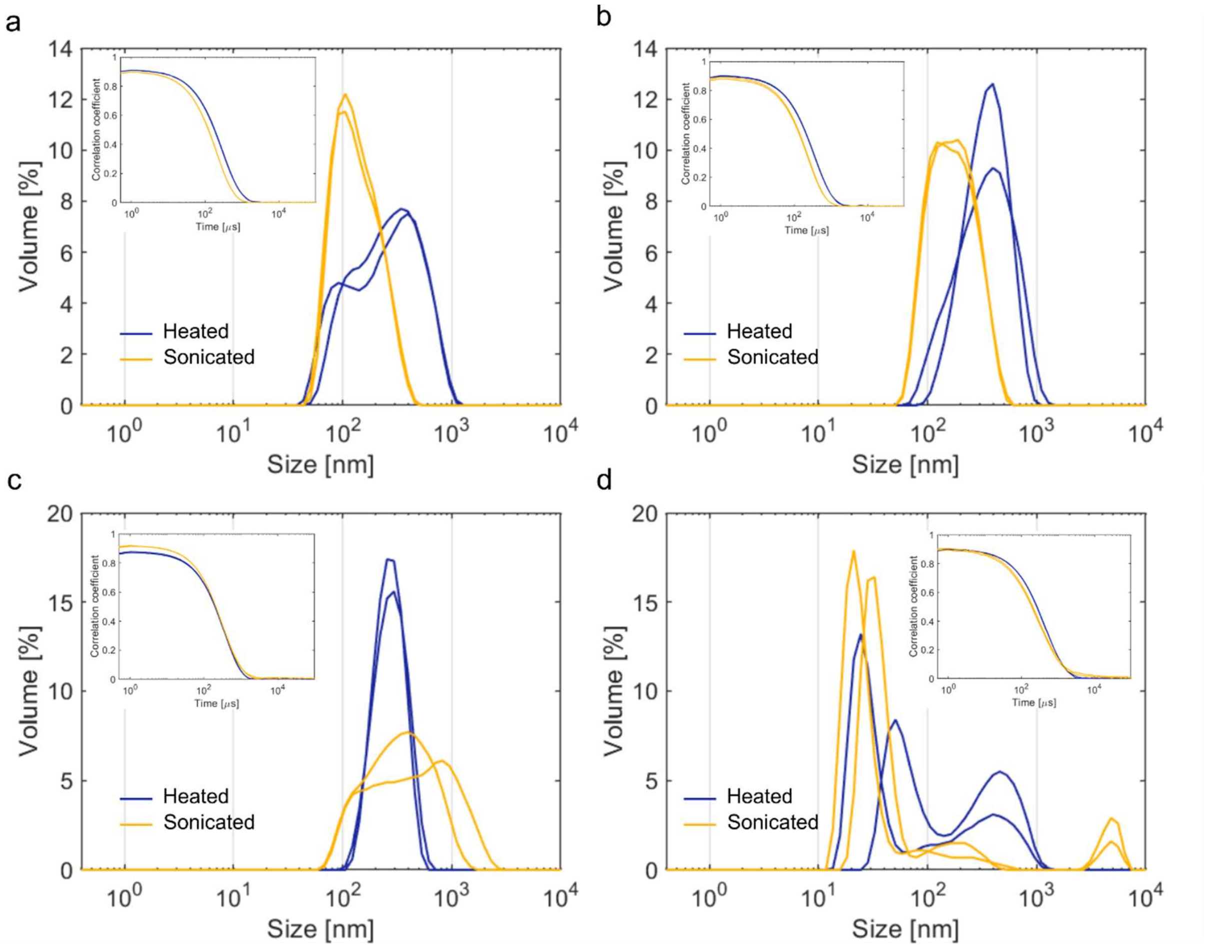

Supplementary Figure 2 Size distributions of the SPI particles determined through dynamic light scattering (DLS). SPI was dissolved in water (a), pH 10 NaOH (b), pH 2 HCl (c) or 30 v/v% acetic acid (d). Samples were prepared either through heating (blue) or ultrasonication (yellow). Inset shows the raw correlogram.

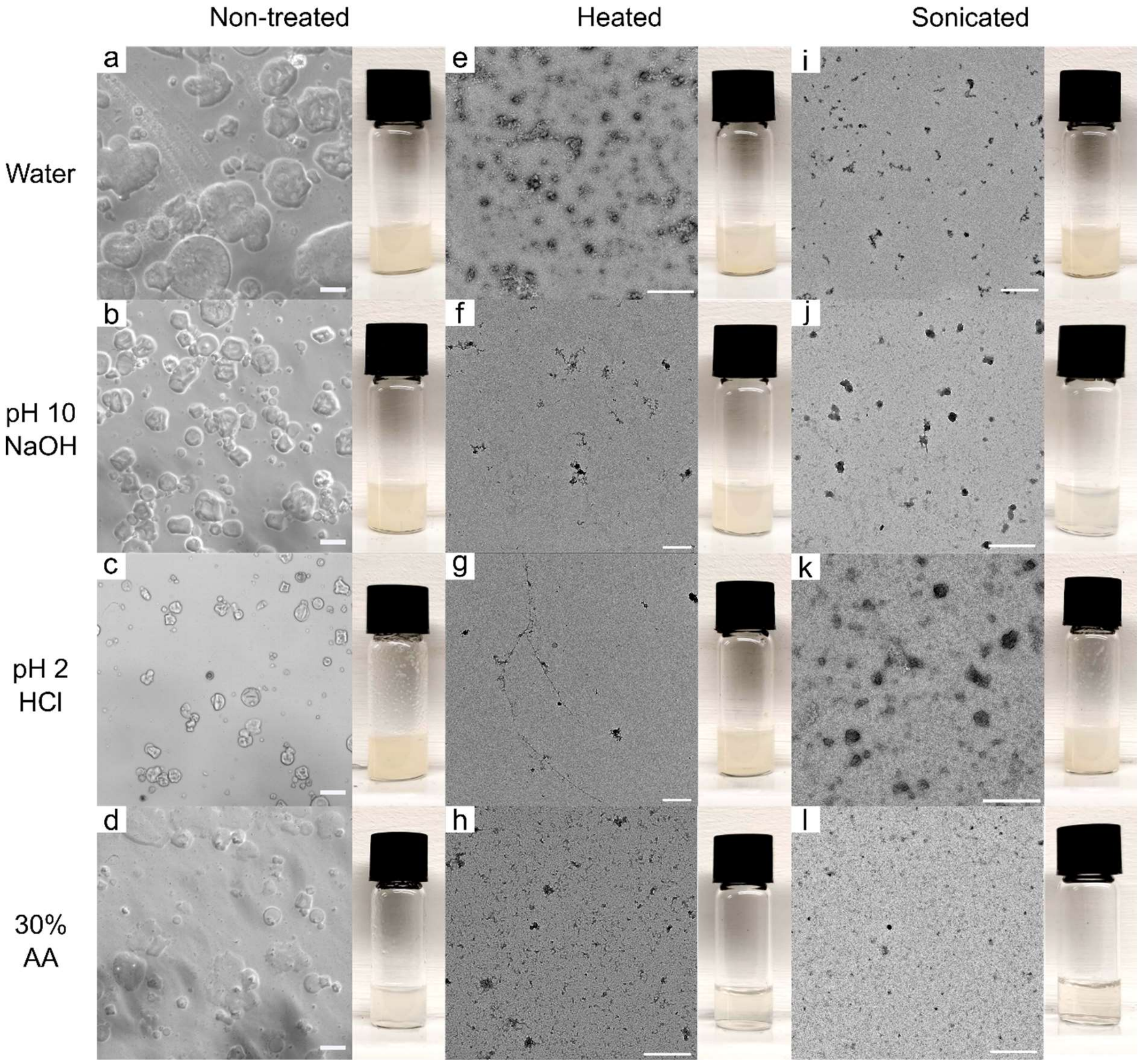

Supplementary Figure 3 Optical images (a-d) and transmission electron microscopy (TEM) images (e-l) of SPI dispersions prepared with water (a,e,i), pH 10 NaOH (b,f,j), pH 2 HCl (c,g,k) and 30 v/v% acetic acid (d,h,l). Dispersions were used without any treatments (a-d) or treated with heat (e-h) or ultrasonication (i-l). The scale bars are 50 μm for optical images (a-d), and 1 μm for e and 500 nm for the rest TEM images (f-l).

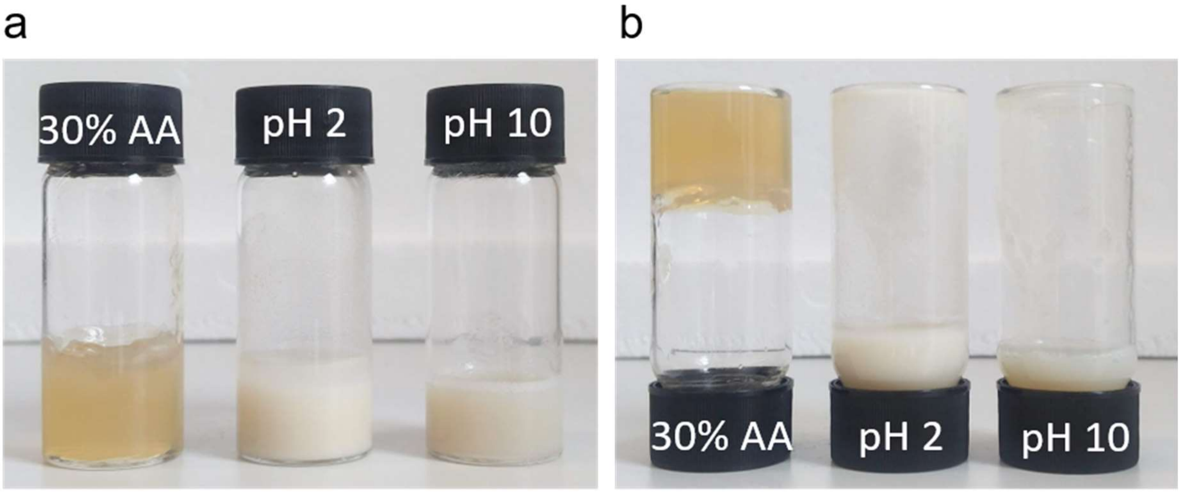

Supplementary Figure 4 Optical images of 10 w/v% SPI dispersions prepared in different solvents with ultrasonication treatment (a) SPI dispersions in 30 v/v% acetic acid (left), pH 2 HCl (middle) and pH 10 NaOH after 30 min of sonication (right). (b) Inverted glass vials of (a) show that the hydrogel was formed in the 30 v/v% acetic acid sample.

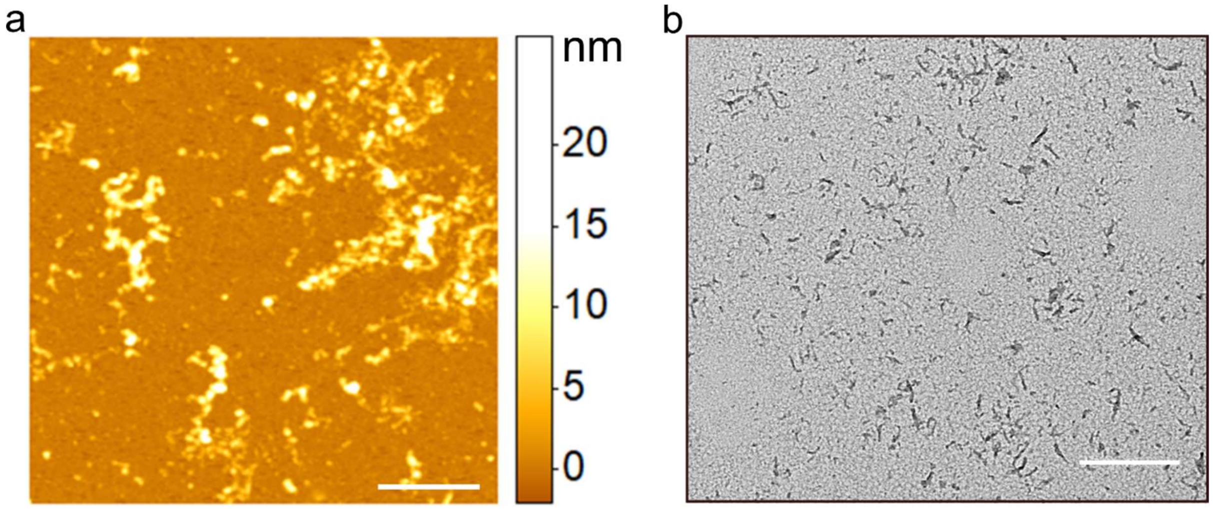

Supplementary Figure 5 Atomic force microscopy (AFM) image (a) and TEM image (b) of the fibrillar aggregates formed within the hydrogel. For imaging, hydrogel was heat-melted at 95°C and diluted in 30 v/v% acetic acid. Scale bars are 500 nm.

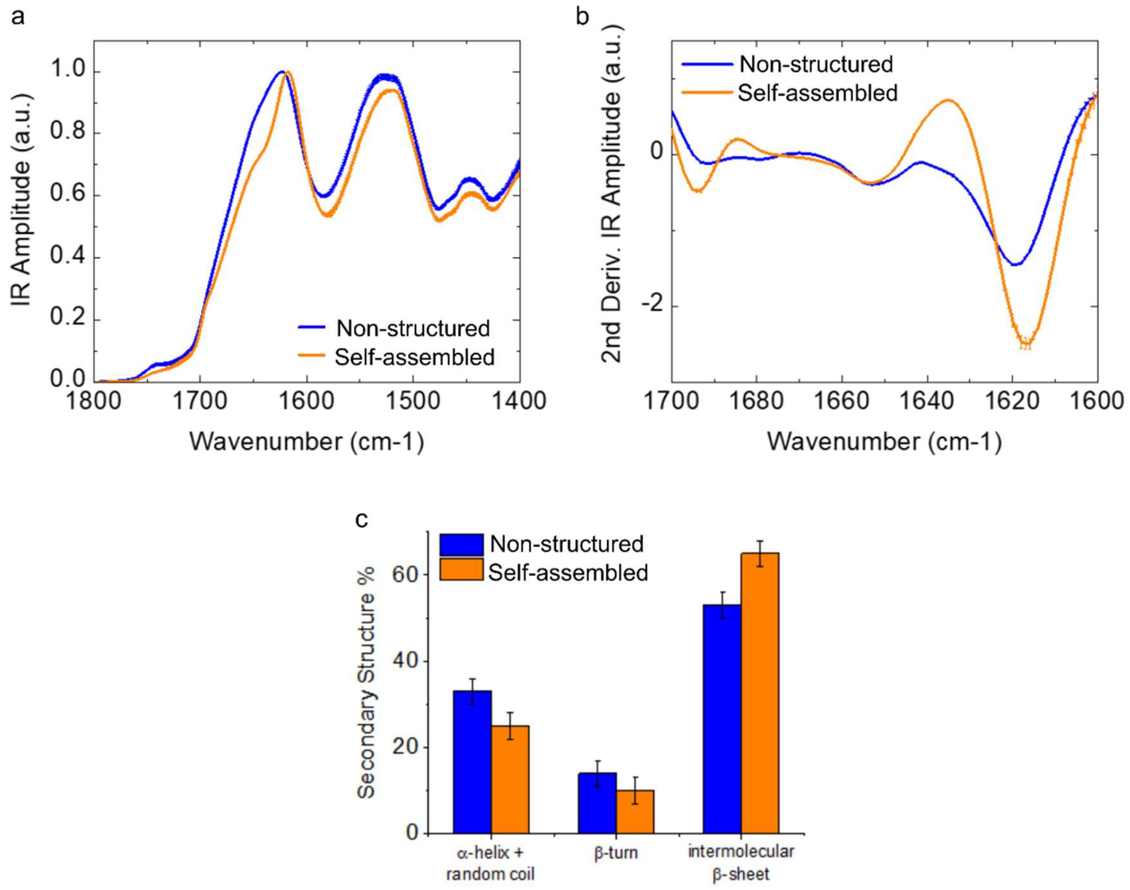

Supplementary Figure 6 Structural analysis of proteins through ATR-FTIR for the non-structured film (prepared with pH 10 NaOH and heated at 95°C for 30 min) and the self-assembled film. (a) FTIR spectra of the films. (b) Second derivatives of amide I band calculated from the FTIR spectra. (c) Quantification of secondary structure content calculated from the second derivatives of amide I band. The indicated error bars are the s.d. of the average of three different spectra, each one is co-average of 256 scans.

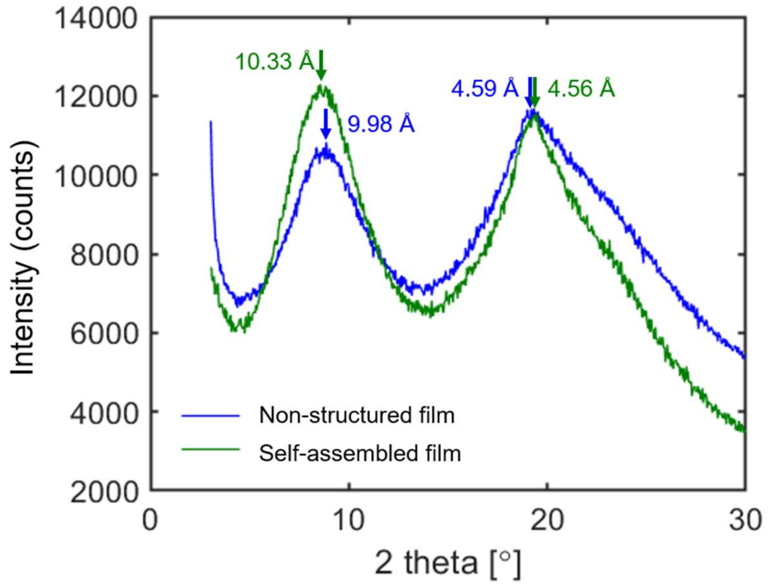

Supplementary Figure 7 XRD analysis of original SPI powder (blue) and the self-assembled film (green). It was confirmed that the amount of intermolecular β-sheet (spacing ca 10 Å)[1] was increased in the self-assembled film.

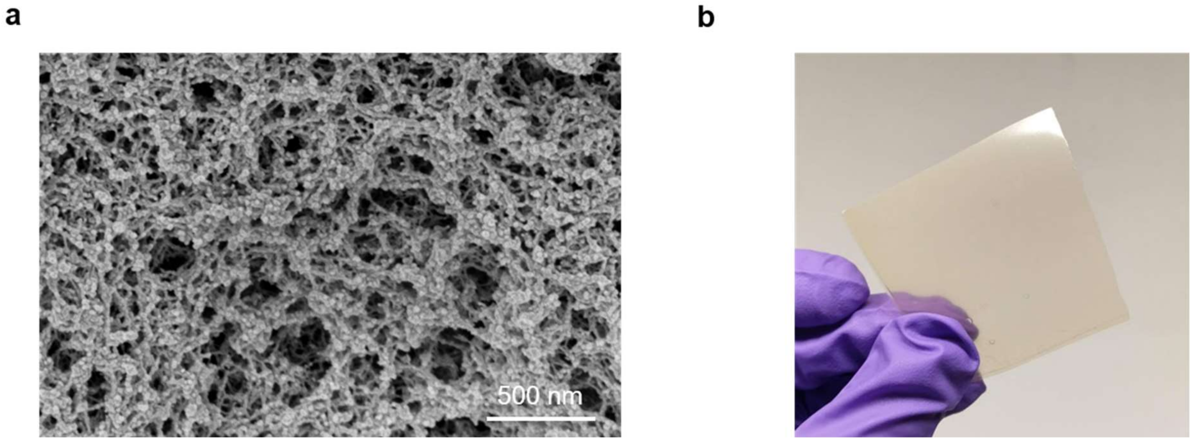

Supplementary Figure 8 Processing of pea protein isolate (Cambridge Commodities ltd). The solution was prepared using 30 v/v% acetic acid and treated with ultrasonication. (a) SEM image of the pea protein hydrogel. The sample was prepared through critical point drying process. (b) Optical image of the pea protein film generated through solvent-casting of sonicated pea protein solution.

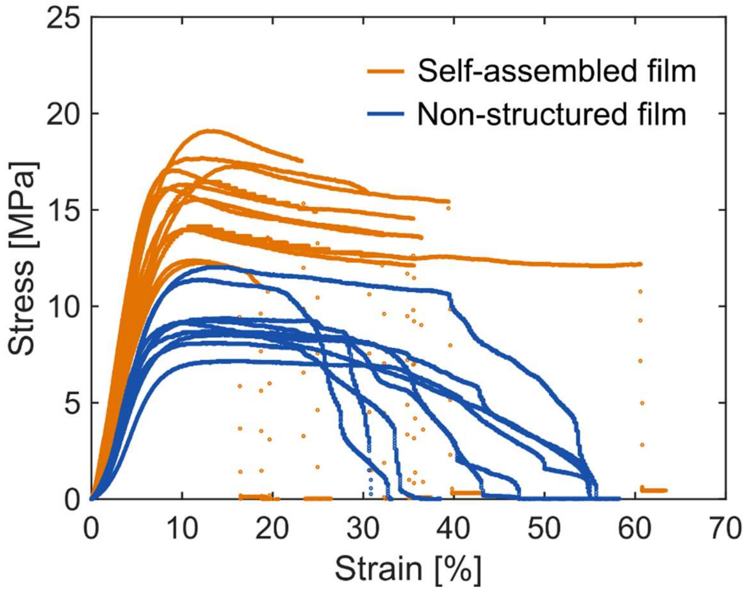

Supplementary Figure 9 Tensile tensing curves for the non-structured film (blue) and self-assembled film (orange). Measurements were repeated at least 9 times.

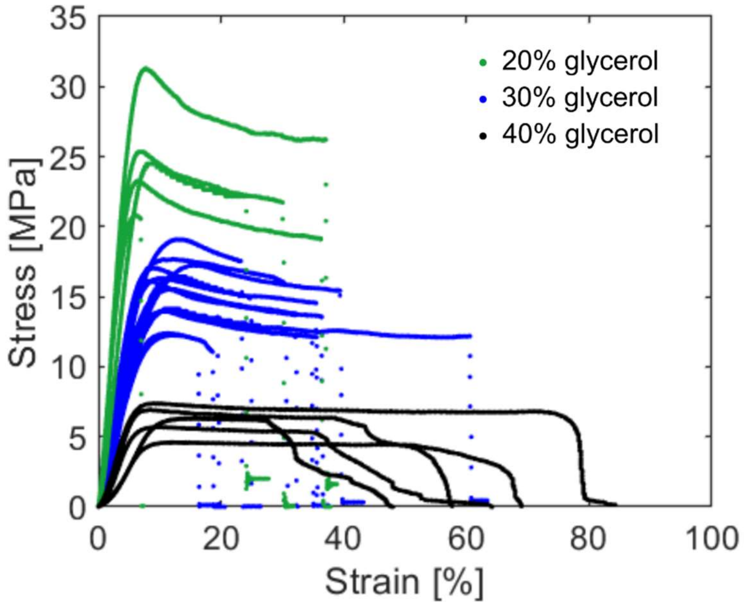

Supplementary Figure 10 Strain-stress curves for films containing different amount of glycerol, 20 w/w% (green), 30 w/w% (blue) and 40 w/w% (black).

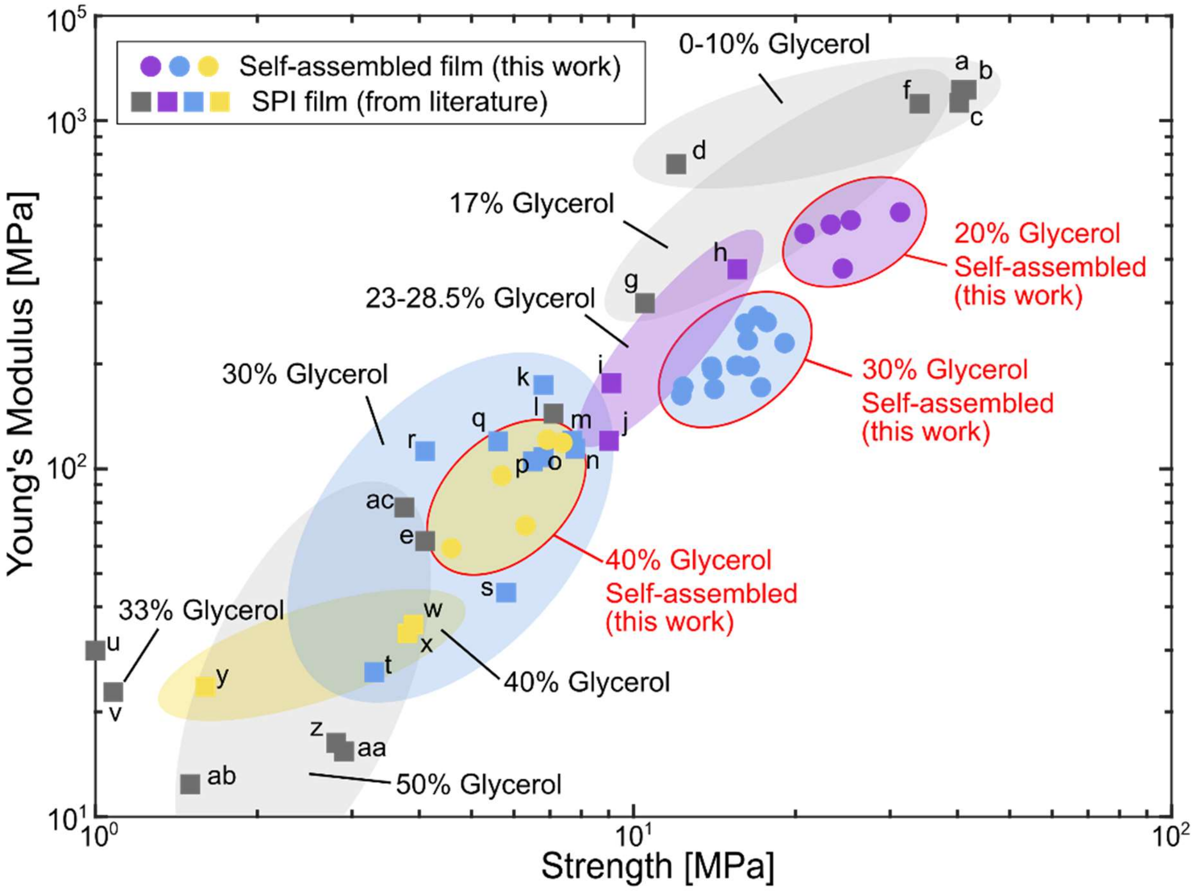

Supplementary Figure 11 Mechanical properties of the dried SPI film in comparison to previously reported SPI films with different amount of glycerol (see Supplementary Table 3 for references). The previously reported values are divided into groups with similar concentration of glycerol, 23-28.5 w/w% (purple), 30 w/w% (blue) and 40 w/w% (yellow).

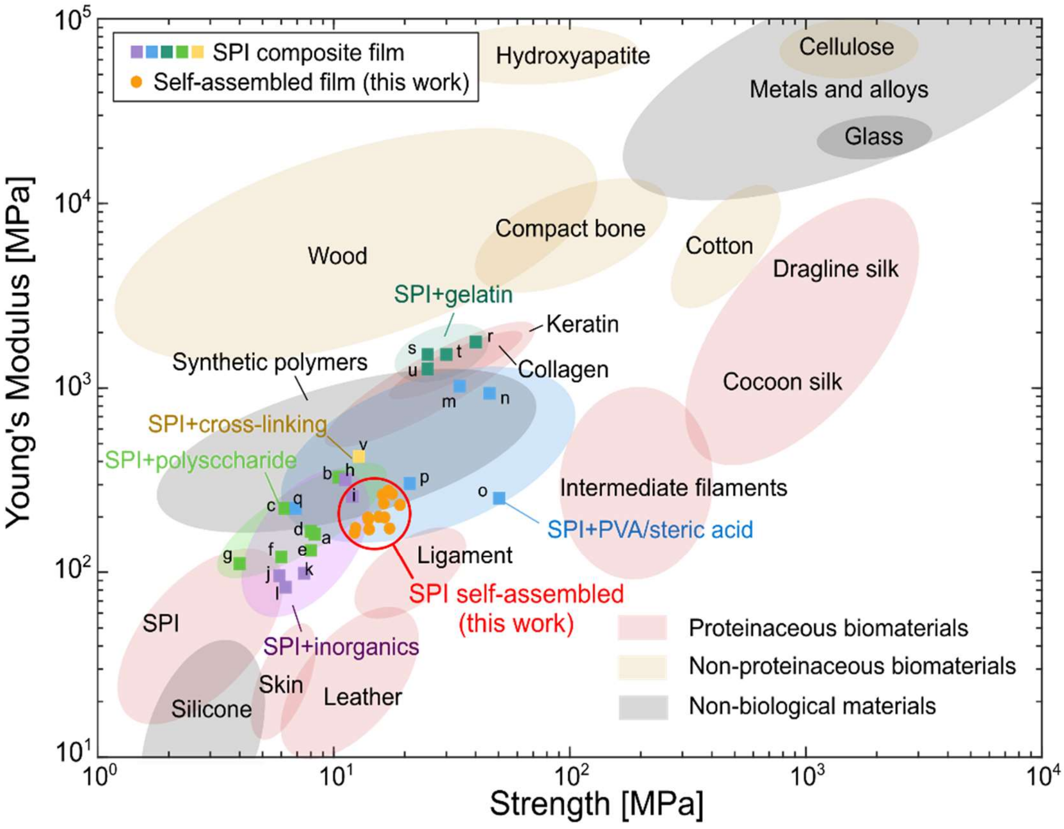

Supplementary Figure 12 Mechanical properties of the dried SPI film in comparison to previously reported biomaterials and engineered materials as well as SPI composite materials (see Supplementary Table 4 for the details of references).

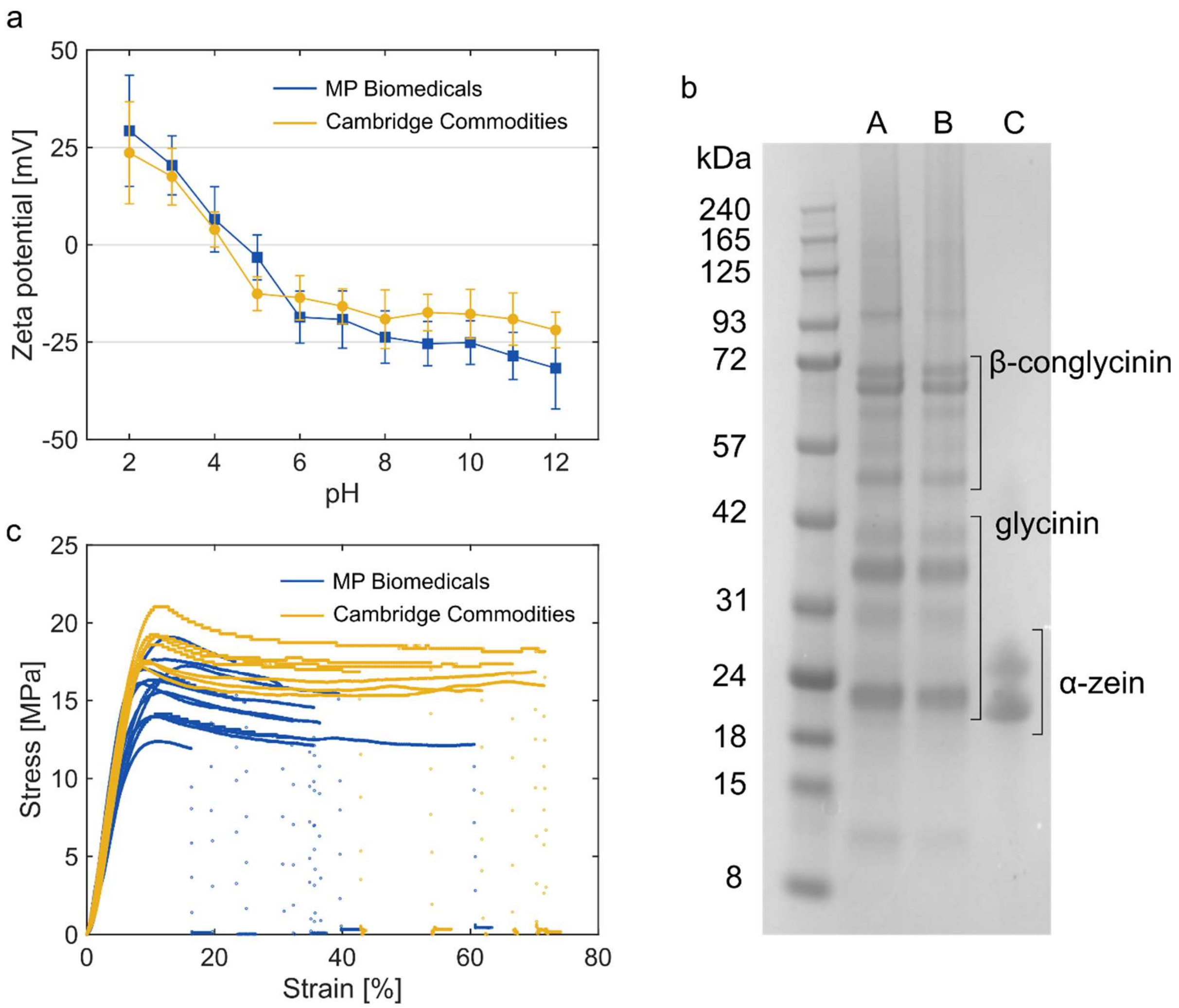

Supplementary Figure 13 Characterization of SPIs obtained from different manufacturers. The SPI from MP Biomedicals contains 92.0% of proteins, 6.0% of moisture and 4.1% of ash. The purity of SPI from Cambridge Commodities is reported to be 90%. (a) Zeta potential measurements for SPIs obtained from MP Biomedicals and Cambridge commodities. Data are shown as mean ± s.d.; n = 3 independent measurements. (b) SDS-PAGE electrophoretogram of SPI from MP Biomedicals (A), SPI from Cambridge Commodities (B) and corn zein, used for coating application (Figure 4e) (C)[2,3]. (c) Tensile testing of the films prepared from different SPIs.

Supplementary Table 1 Summary of the reported opacity of soy protein film and its comparison to our film.

Film details	Opacity (Abs/mm)	Reference
Self-assembled film (30% glycerol)	0.654	This work<br>Absorbance at 550 nm
Non-structured film (30% glycerol)	2.74	
SPI (33% glycerol)	1.35 ± 0.20	Gonzalez et. al. (2013)[4]<br>Absorbance at 400-800 nm
SPI with 50% PLA (33% glycerol)	0.71 ± 0.12	
SPI (30% glycerol, casting)	8.71	Garrido et. al. (2016)[5]<br>Absorbance at 600 nm
SPI (30% glycerol, compression)	1.16	
SPI (9% glycerol)	1.326 ± 0.15	Cao et. al. (2007)[6]<br>Absorbance at 500 nm

Supplementary Table 2 Summary of references used for plant protein-based materials in Fig.3b.

Materials	Reference
Starch	Jimenez et. al. 2012[7]
Peanut protein	Reddy et. al. 2013[8]<br>Zink et. al. 2016[9]<br>Shit et. al. 2014[10]
Corn zein	
Soy protein	
Wheat gluten	
Whey protein	Schmid et. al. 2018[11]

Supplementary Table 3 References for the previously reported mechanical properties of soy protein films shown in Fig.3c and Supplementary Figure 10.

Fig. S11	Fig.3c	Amount of glycerol	Reference
a		9% glycerol	Zhang et. al. 2001[12]
b,c		0/2% glycerol	Su et. al. 2007[13]
d		10% glycerol	Cao et. al. 2007[6]
e		0% glycerol	Rampon et. al. 1999[14]
f		17% glycerol	Zhang et. al. 2001[12]
g		17% glycerol	Vaz et. al. 2003[15]
h,i		23/28.5% glycerol	Zhang et. al. 2001[12]
j		23% glycerol	Lodha et. al. 2005[16]
k	a	30% glycerol	Ai et. al. 2007[17]
l		33% glycerol	Zhang et. al. 2001[12]
m,n,r	b,c,d	30% glycerol	Guerrero et. al. 2010[18]
o	e	30% glycerol	Zheng et. al. 2007[19]
p	f	30% glycerol	Zheng. et. al. 2009[20]
q	g	30% glycerol	Zheng et. al. 2003[21]
s	i	30% glycerol	Wang et. al. 2006[22]
t	h	30% glycerol	Lu et. al. 2004[23]
u		33% glycerol	Chen et. al. 2008[24]
v		33% glycerol	González et. al. 2013[4]
w,x,y		40% glycerol	Guerrero et. al. 2010[18]
z,aa,ab		50% glycerol	
ac		50% glycerol	Li et. al. 2017[25]

Supplementary Table 4 References for the previously reported mechanical properties of soy protein composite films shown in Supplementary Figure 11.

A	30% cellulose whiskers	Wang et. al. 2006[22]
b	2% pea starch nanocrystals	Zheng et. al. 2009[20]
c	25% chitin	Zheng et. al. 2003[21]
d,f,g	11.3 – 20.2% cellulose nanofibers	Chen et. al. 2008[24]
e	20% chitin whiskers	Lu et. al. 2004[23]
h	4% SiO2	Ai et. al. 2007[17]
i	0.25% carbon nanotubes	Zheng et. al. 2007[19]
j,k,l	Graphene + cellulose	Li et. al. 2017[25]
m,n,o,p	10/20% PVA	Su et. al. 2007[13]
q	25% steric acid	Lodha et. al. 2005[16]
r,s,t,u	20-80% gelatin	Cao et. al. 2007[6]
v	UV cross-linking	Vaz et. al. 2003[26]

Supplementary References

1. Fitzpatrick, A. W. P. *et al.* Atomic structure and hierarchical assembly of a cross-β amyloid fibril. *Proc. Natl. Acad. Sci. U. S. A.* **110**, 5468–5473 (2013).

2. Chen, N., Zhao, M., Chassenieux, C. & Nicolai, T. Data on the characterization of native soy globulin by SDS-Page, light scattering and titration. *Data Br.* **9**, 749–752 (2016).

3. Díaz-Gómez, J. L., Ortíz-Martínez, M., Aguilar, O., García-Lara, S. & Castorena-Torres, F. Antioxidant activity of Zein hydrolysates from zea species and their cytotoxic effects in a hepatic cell culture. *Molecules* **23**, 312 (2018).

4. González, A. & Alvarez Igarzabal, C. I. Soy protein - Poly (lactic acid) bilayer films as biodegradable material for active food packaging. *Food Hydrocoll.* **33**, 289–296 (2013).

5. Garrido, T., Leceta, I., Cabezudo, S., Guerrero, P. & de la Caba, K. Tailoring soy protein film properties by selecting casting or compression as processing methods. *Eur. Polym. J.* **85**, 499–507 (2016).

6. Liao, P. *et al.* Preparation and physical properties of soy protein isolate and gelatin composite films. *Gongneng Cailiao/Journal Funct. Mater.* **40**, 291–294 (2009).

7. Jiménez, A., Fabra, M. J., Talens, P. & Chiralt, A. Edible and Biodegradable Starch Films: A Review. *Food Bioprocess Technol.* **5**, 2058–2076 (2012).

8. Reddy, N. & Yang, Y. Thermoplastic films from plant proteins. *J. Appl. Polym. Sci.* **130**, 729–738 (2013).

9. Zink, J., Wyrobnik, T., Prinz, T. & Schmid, M. *Physical, chemical and biochemical modifications of protein-based films and coatings: An extensive review. International Journal of Molecular Sciences* **17**, (2016).

10. Shit, S. C. & Shah, P. M. Edible Polymers: Challenges and Opportunities. *J. Polym.* **2014**, 1–13 (2014).

11. Schmid, M. & Müller, K. Whey protein-based packaging films and coatings. *Whey Proteins From Milk to Med.* **661**, 407–437 (2018).

12. Zhang, J., Mungara, P. & Jane, J. Mechanical and thermal properties of extruded soy protein sheets. *Polymer (Guildf).* **42**, 2569–2578 (2001).

13. Su, J. F., Huang, Z., Liu, K., Fu, L. L. & Liu, H. R. Mechanical properties, biodegradation and water vapor permeability of blend films of soy protein isolate and poly (vinyl alcohol) compatibilized by glycerol. *Polym. Bull.* **58**, 913–921 (2007).

14. Rampon, V., Robert, P., Nicolas, N. & Dufour, E. Protein structure and network orientation in edible films prepared by spinning process. *J. Food Sci.* **64**, 313–316 (1999).

15. Vaz, C. M. *et al.* Casein and soybean protein-based thermoplastics and composites as alternative biodegradable polymers for biomedical applications. *J. Biomed. Mater. Res. - Part A* **65**, 60–70 (2003).

16. Lodha, P. & Netravali, A. N. Thermal and mechanical properties of environment-friendly ‘green’ plastics from stearic acid modified-soy protein isolate. *Ind. Crops*

Prod. **21**, 49–64 (2005).

17. Ai, F., Zheng, H., Wei, M. & Huang, J. Soy protein plastics reinforced and toughened by SiO2 nanoparticles. *J. Appl. Polym. Sci.* **105**, 1597–1604 (2007).

18. Guerrero, P. & De La Caba, K. Thermal and mechanical properties of soy protein films processed at different pH by compression. *J. Food Eng.* **100**, 261–269 (2010).

19. Zheng, H., Ai, F., Wei, M., Huang, J. & Chang, P. R. Thermoplastic soy protein nanocomposites reinforced by carbon nanotubes. *Macromol. Mater. Eng.* **292**, 780–788 (2007).

20. Zheng, H., Ai, F., Chang, P. R., Huang, J. & Dufresne, A. Structure and Properties of Starch Nanocrystal-Reinforced Soy Protein Plastics. *Polym. Compos.* **30**, 474–480 (2009).

21. Zheng, H., Tan, Z., Zhan, Y. R. & Huang, J. Morphology and Properties of Soy Protein Plastics Modified with Chitin. *J. Appl. Polym. Sci.* **90**, 3676–3682 (2003).

22. Wang, Y., Cao, X. & Zhang, L. Effects of cellulose whiskers on properties of soy protein thermoplastics. *Macromol. Biosci.* **6**, 524–531 (2006).

23. Lu, Y., Weng, L. & Zhang, L. Morphology and properties of soy protein isolate thermoplastics reinforced with chitin whiskers. *Biomacromolecules* **5**, 1046–1051 (2004).

24. Chen, G. & Liu, H. Electrospun cellulose nanofiber reinforced soybean protein isolate composite film. *J. Appl. Polym. Sci.* **110**, 641–646 (2008).

25. Li, K., Jin, S., Han, Y., Li, J. & Chen, H. Improvement in functional properties of soy protein isolate-based film by cellulose nanocrystal-graphene artificial nacre nanocomposite. *Polymers (Basel).* **9**, (2017).

26. Vaz, C. M., De Graaf, L. A., Reis, R. L. & Cunha, A. M. Effect of crosslinking, thermal treatment and UV irradiation on the mechanical properties and in vitro degradation behavior of several natural proteins aimed to be used in the biomedical field. *J. Mater. Sci. Mater. Med.* **14**, 789–796 (2003).
