# Supplementary material for: Controlled self-assembly of plant proteins into high-performance multifunctional nanostructured films
Source: Nat Commun. 2021 Jun 10;12:3529. doi: 10.1038/s41467-021-23813-6 (PMC8192951; doi:10.1038/s41467-021-23813-6)
Supplement: Supplementary file 2 — Description of Additional Supplementary Files [file 41467_2021_23813_MOESM2_ESM.docx]

**Description of Supplementary Movie 1:**

Water absorption of protein-coated paperboards visualised using cobalt chloride.
